# Reconstructing spatial pattern of historical cropland in karst areas of Guizhou, Southwest China

**DOI:** 10.1038/s41598-022-26793-9

**Published:** 2022-12-27

**Authors:** Liuying Yang, Cuiwei Zhao, Shulin Jiao, Shuang Li, Lei Wang, Yinjiu Li

**Affiliations:** 1 School of Geography and Environmental Science/Karst Research Institute, Guizhou Normal University, No. 116, North Baoshan Road, Guiyang, 550001 China; 2 The State Key Laboratory Incubation Base for Karst Mountain Ecological Environment of Guizhou Province, Guiyang, 550001 China; 3 Henan Jinchen Information Technology Co., Ltd., Zhengzhou, 450000 China

**Keywords:** Environmental social sciences, Sustainability

## Abstract

Karst regions are exceptionally responsive to global change with their harsh natural environment, fragile ecology, and acute human-land conflicts. The reconstruction of cropland spatial pattern in karst areas during the historical period is typical for studying human-land relations in karst areas and has important practical significance for climate study. The ecological environment changes at regional and global scales, primarily to provide essential data and a theoretical basis for studying the inverse evolution of rock desertification in karst areas. Guizhou province, a typical karst area, was selected as the research area in 1820. Based on the correction of historical population data and cropland data, a reconstruction model of cropland spatial pattern in karst areas during the historical period was constructed by selecting factors such as elevation, slope, soil types, organic matter content, climatic productivity potential, distance to river and distance from settlements to reconstruct the spatial pattern distribution of cropland in 1820 of Guizhou. The results show that the data on cropland recorded in Guizhou during the Qing dynasty is too low, mainly due to Yin-Ni and the policy of Tu-Di-Mian-Ke. In 1820, the total area of revised cropland in Guizhou was 1,851,792 hm^2^, with the highest proportion of 14.32% in Dading Fu and the lowest in Songtao Ting at 1.6%. Only 30% of the grid in Guizhou has a cropland distribution. It is mainly concentrated in the central part of Qianzhong District (Anshun and Guiyang Fu), the southern part of Qianbei District (Pingyue Fu and southern Zunyi Fu), the western part of Qiandongnan District, the central and eastern parts of the Qiandongbei District. The overall average reclamation rate of land in Guizhou is 10.93%, the highest reclamation intensity in Qianzhong District, with 8.5% of grids ≥ 50%, and the smallest in Qianxinan District, with only 1.65% of grids ≥ 50%. The analysis is validated by comparing the reconstruction model and the reconstruction results. It can be seen that the reconstruction model and research results of this paper can more objectively reflect the distribution of cropland in karst areas during the historical period, and the reconstruction model is suitable for karst areas with low productivity levels.

## Introduction

Land use/cover change (LUCC) caused by human activities is an essential factor affecting climatic and ecosystem changes on regional and global scales. It has become the focus of global ecological and environmental change research^1–5^. In 1995, the International Geosphere-Biosphere Programme (IGBP) and the International Human Dimensions Programme on Global Environmental Change (IHDP), two international organizations, regarded the Land Use/Cover Change Scientific Research Programme as an essential part of global change research. One of the critical issues is how human activities have changed land cover over the last 300 years, emphasising the need to reconstruct the detailed history of past land-use change by various means, thus launching a wave of research on LUCC in the historical periods^6–13^. In recent years, global land-use cover reconstruction research has made positive progress in the past 300 years, driven by studies such as the BIOOME300 project, GCTE, GLP, iLEAPS and PAGES, Etc^14,15^. With the continuous development of research on the reconstruction of LUCC in historical periods, scholars have conducted extensive research on the reconstruction of land use data with spatial attributes in the historical periods. Reconstructing the spatial and temporal distribution patterns of cropland, forest land and construction land at global, national and regional scales^10,16–24^.

Cropland is one of the most significant land types where human activities affect LUCC. Its characteristics and dynamics are vital in studying LUCC in a specific region. The study of the reconstruction of cropland patterns reveals the evolution of human-land relations in historical times. It provides a theoretical basis for studying processes that simulate past climatic and ecological changes^25^. From the perspective of methodology, the historical reconstruction methods of cropland gradually developed from qualitative analysis relying on historical documents to quantitative analysis based on multi-source data and interdisciplinary knowledge application. To some extent, the current research results reveal the spatial and temporal patterns of cropland change in China during the historical period and provide data support for the analysis of the driving factors of land use change, global environmental change and the terrestrial carbon cycle. However, some studies only used surrogate factors to correct the primary data, lacking accurate literature research and a reasonable revision process. Current studies are mainly carried out on the global, national or provincial levels. China's studies mainly focus on the northeast and traditional agricultural areas, the Qinghai-Tibet Plateau and Taiwan^24,26–30^, with relatively few studies on historical LUCC for karst areas. In the accuracy of the reconstruction, the spatial grids set by current studies are often too large, mostly historical land use reconstruction studies at 1 km × 1 km or coarser spatial resolution. They lack historical land use reconstruction studies at a higher spatial resolution, which cannot reflect the actual land use characteristics in typical regions, especially in karst areas.

Karst areas have a harsh natural environment, fragile ecology and sharp human-land conflicts responsive to global change. It is characterized by a sensitive response to environmental change, weak resistance to disturbance and surface fragmentation. Environmental change in the region will intensify as human interference with the natural environment increases and amplifies the effect of environmental change. Due to the fragmentation of cropland in karst areas and the small scale of arable plots, the reconstruction results at a larger grid scale cannot truly reflect the spatial distribution pattern of cropland in karst areas during the historical period. It is also difficult to accurately explore the cropland characteristics in the karst mountains area. Thus, it is crucial to carry out a study on reconstructing the spatial distribution pattern of cropland in karst areas at high resolution during the historical period. Because of this, our study constructed a gridded reconstruction model of the spatial distribution pattern of cropland in the historical period with a higher resolution (90 m × 90 m) than previous studies, based on the revision of the historical population data and historical cropland data in the register. At the same time, our study also verified the rationality of the model and the accuracy of the reconstruction results by using the comparative validation method.

Guizhou is located in the ecologically fragile and sensitive karst area of southwest China. The area has complex and diverse landscape types, high mountains and steep slopes, fragmented cropland and insufficient arable resources; with a significant population change over the past 300 years, the conflict between people and land is acute^31,32^. Guizhou, a typical karst region, was chosen as the study area. The 25th year of Jiaqing (1820) during the Qing Dynasty was used as the study point to reconstruct the pattern of cropland during the historical period. It is not only typical for the study of human-land relations in karst areas but also has important practical significance for the study of regional climate and ecological environment changes. In particular, it can provide essential data and a theoretical basis for studying stone desertification inversion in karst areas.

## Study area

Guizhou is located in the southwest of China, in the northeast of the Yunnan-Guizhou Plateau, in the overland between the plateau of eastern Yunnan and the hills of western Hunan. It is a subtropical karst plateau mountainous region rising between the Sichuan basin and the hills of Guangxi and western Hunan^33^. The terrain is undulating, high in the west and low in the east. The average altitude is 1100 m, the highest is 2900 m, and the lowest is only 137 m, with a total land area of about 17.62 × 10^4^ km^2^. It is a typical karst landform area with typical karst landscape development, extensive carbonate rock formation and a fragile ecological environment. Karst landscapes account for 61.9% of the province's total area. It is the only mountainous agricultural province in the country dominated by karst landscapes and has no plains.

Guizhou has a long history of agricultural production and agricultural development. According to archaeological data, primitive agriculture emerged in Guizhou during the Neolithic Age, entering the primitive farming stage and engaging in rice farming and other production activities^34^. With the succession of dynasties, primitive agriculture developed for a certain period, and farming had a particular scale and level. However, due to the influence of backward production technology, the agricultural economy of Guizhou was far behind that of the Central Plains.

During the Ming Dynasty, with the improvement of transportation, large-scale Jun-Shi-Tun-Tian, the policy of the Diao-Bei-Tian-Nan and the Yi-Min-Shi-Bian, more advanced production techniques and experience were brought in, and the experience was spread in the construction of weirs, ponds, canals and the use of oxen for ploughing^35^. With the increase in population and advanced farming tools, Guizhou's land was further reclaimed, and the area under cultivation expanded.

The Qing government took a series of measures to restore agricultural production to consolidate its rule in the border areas and stabilize the development of Guizhou. In order to restore and develop the social economy of Guizhou, which had been destroyed by the war, a series of policies on land reclamation, Tun-Tian and the policy of Mian-Ke, were introduced during the Kangxi period. During the Yongzheng and Qianlong periods, they continued to be implemented as a basic state policy. During the Yongzheng period, about 43,844 Qing mu of new fields were reclaimed, and a total of 978,442 Qing mu of farmland was reclaimed during the Qianlong period. The reclamation of farmland during the Shunzhi, Kangxi, Yongzheng and Qianlong dynasties significantly accelerated the development of agriculture in Guizhou.

Based on the structure of crops, the level of development of agricultural production, climatic conditions, topographical features and similarities in the agricultural production environment in Guizhou during the Qing Dynasty, and on the premise of maintaining the integrity of certain levels of administrative boundaries, Guizhou in 1820 was divided into six agricultural districts. They are Qianbei, Qiandongbei, Qiandongnan, Qianxinan, Qianxibei and Qianzhong^36–38^.

**Figure 1:**
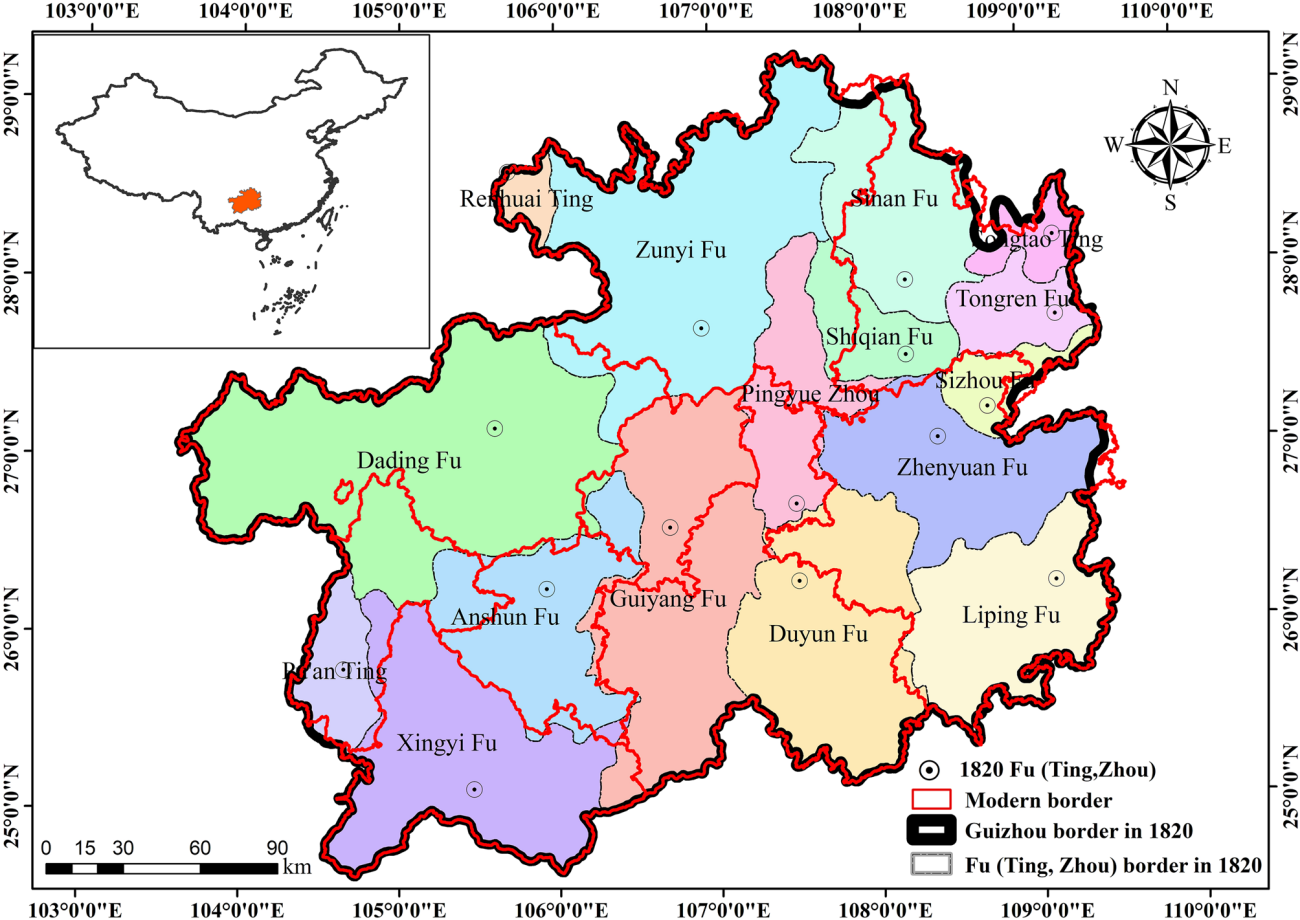
Location of the study area, ancient and modern administrative boundaries(map created using ArcGIS 10.8).

The administrative boundaries of Guizhou in 1820 were essentially the same as those of modern Guizhou. For research and data processing, the administrative boundaries of Guizhou in 1820 were used as the outer boundaries of the study area, and the administrative boundaries of its 16 internal Fu (Ting) were used as the county boundaries of the study area (Fig. 1).

## Data sources and processing

### Basic data

The 90 m × 90 m DEM data was derived from the Geospatial Data Cloud (http://www.gscloud.cn), and the slope data required for the study were extracted from ArcGIS. Soil, climate production potential data, and mean ≥ 10° annual accumulation temperature data were provided by Data Sharing Infrastructure of Earth System Science (http://www.geodata.cn.), in which the raw mean ≥ 10° annual accumulation temperature data with a grid resolution of 500 m × 500 m were resampled to a 90 m × 90 m grid. Data on rivers, water bodies, and geographic data on historical village settlements and administrative boundaries from 1820 were used, using line and surface data from the Harvard University CHGIS database^39^.

### Population data correction

Guizhou is an ethnic minority region. Although the Gai-Tu-Gui-Liu policy was implemented in Yongzheng and Qianlong periods, the Tu-Si system has yet to be completely eradicated in Guizhou. Many chief officials and deputy chiefs officials remain^40^. Until 1820, the population data was still underreported, and the population data in the register was relatively low. Therefore, the recorded population data should be revised before reconstructing cropland spatial patterns in the historical period.

Revised population data of Guizhou in 1820 (Table 1), citing the revised data in the “*Population History of China (Vol. 5): Qing Dynasty*”^41^. Chinese scholar Cao Shuji revised the data based on historical materials of the Qing Dynasty, such as local chronicles of each prefecture, relevant data on the population of ethnic minorities in Guizhou and their proportion published in 1985, and has a high degree of authority and credibility.

**Table 1:** Correction of population data.

Name	Population (thousands)	Proportion (%)	Name	Population (thousands)	Proportion (%)
Guiyang Fu	920	12.3	Sinan Fu	639	8.6
Anshun Fu	788	10.5	Songtao Ting	120	1.6
Dading Fu	1071	14.3	Tongren Fu	131	1.8
Pu'an Ting	140	1.9	Sizhou Fu	126	1.7
Xingyi Fu	437	5.9	Pingyue Fu	373	5.0
Shiqian Fu	159	2.1	Zhenyuan Fu	583	7.8
Zunyi Fu	1064	14.2	Duyun Fu	500	6.7
Renhuai Ting	91	1.2	Liping Fu	336	4.5

### Cropland data correction

#### Cropland data in the register

The recorded cropland data were mainly obtained in two ways: one is obtained from local chronicles, and the other is obtained from publicly published materials and research results of experts and scholars^42,43^. The recorded cropland data is shown in Table 2.

**Table 2:** Cropland data in the register.

Name	Area (km^2^)	Quantity (Qing mu)	Name	Area (km^2^)	Quantity (Qing mu)
Guiyang Fu	17,700	267,603	Sinan Fu	12,300	104,367
Anshun Fu	12,900	252,738	Songtao Ting	2,400	22,303
Dading Fu	17,100	230,591	Tongren Fu	3,000	55,786
Pu'an Ting	4,650	39,112	Sizhou Fu	2,700	57,202
Xingyi Fu	9,600	86,826	Pingyue Fu	6,300	210,023
Shiqian Fu	3,000	59,494	Zhenyuan Fu	11,700	206,148
Zunyi Fu	16,200	896,874	Duyun Fu	15,300	99,188
Renhuai Ting	2,700	23,566	Liping Fu	11,100	155,520

Records of historical cropland in China are primarily scattered in local chronicles, taxation, classics and statistical materials, which cannot truly reflect the actual situation of historical cropland due to various problems such as Mu-Zhi, Yin-Ni, false reporting and underreporting^44^. It is necessary to sift through them, revise and verify them to obtain accurate statistical information on historical cropland.

For a long time, knowledge of the actual area of cropland in the southwest during the historical period has been weak, constrained by natural conditions, the accumulation of historical data, ethnic minorities and the research base and other related factors.

Although many official archives left a series of land figures, there is a considerable discrepancy between the data on cropland contained in Qing dynasty registers and current data on the area of cropland^45,46^. The recorded cropland data during the Qing Dynasty is too low.

As can be seen from Table 3, the cropland figures for Guizhou during the Qing dynasty were basically around one or two million Qing mu, with a significant increase in population but a minor increase in cropland, and the number of field mu per capita was only a few mu.

**Table 3:** Recorded population and cropland data in Guizhou during the Qing Dynasty.

Year	Population data	Cropland quantity (Qing mu)	The per capita number (Qing mu)
1661	13,839	1,074,344	77.63
1685	13,697	959,711	70.07
1724	21,388	1,454,569	68.01
1753	1,418,848	2,573,594	1.81
1765	3,441,656	2,673,062	0.78
1812	5,258,219	2,766,007	0.52
1820	5,348,667	2,767,041	0.52
1851	5,435,590	2,685,400	0.49
1873	4,171,000	2,685,400	0.63
1887	4,804,000	2,765,006	0.57

Relevant statistics show that the cropland statistics in the Republic of China were 1545 × 10^3^ hm^2^ and 4906 × 10^3^ hm^2^ in 1985, which can be inferred as a severe underestimation of the data on cropland contained in the official Guizhou book during the Qing Dynasty. Although there are some problems with the cropland data in the register, there is a close correlation with the actual cropland area. The information required can be uncovered through the data contained in the register and related factors.

#### Unreality analysis of cropland area

Most of the cropland figures recorded in the official archives during the Qing Dynasty in China only represented taxpaying units, not the actual cropland area at that time. The factors that led to the distortion of the data in these archives included the institutional concept of Yuan-e, the Zhe-Mu, the statistical diameter (Mu-Zhi), and the non-institutional Yin-Ni and Mian-Ke. Around these factors, Chinese scholars have done a great deal of revised research on national or regional farmland data of the Song, Ming and Qing dynasties.

1. Yuan-E. An important reason for the large discrepancy between the recorded cropland and the actual cropland data is influenced by the concept of the Yuan-E^45^. However, this was only for areas with good agricultural development and relatively stable taxation, where the actual cropland area exceeded the original area and where Ming dynasty taxation was recorded. Because of the wars in Guizhou during the late Ming and early Qing dynasties, many codices were lost, and it was difficult to collect taxes according to the total amount of the Ming dynasty. The small changes in the figures for cropland in Guizhou after the Qing dynasty were not influenced by the Yuan-E.
2. Zhe-Mu. Zhe-Mu can be achieved by converting different land qualities into taxable acres at a uniform rate for different areas or by setting different tax rates. If the tax rate is divided into different sections, the area does not apply to the Zhe-Mu^47^. During the Qing dynasty, it was common throughout Guizhou to divide the land into upper, middle and lower classes for separate field taxation^43^. It was one of the few regions in the country where taxation at a Zhe-Mu was not practised, and records of cropland were not affected by the Zhe-Mu.
3. Mu-Zhi. During the Qing Dynasty, the Mu-Zhi reported in Guizhou was consistent with the official standards promulgated by the government. While it cannot be ruled out that Guizhou is entirely free of the Mu-Zhi, it is relatively standard and homogeneous in areas governed by the government. Therefore, this study only considers the conversion of Qing-mu to Shi-mu, i.e., 1 Qing-mu = 1.072 present-day acres (Shi-mu)^48^.
4. Yin-Ni. Guizhou is located in a border minority area; a poor agricultural production environment, backward farming conditions, low food production and a considerable amount of minority cropland are not recorded. On the one hand, the Qing government adopted a lenient policy toward land registration in minority areas during Gai-Tu-Gui-Liu. On the other hand, as it was pretty difficult to measure the land in Guizhou at that time, a province-wide measurement of fields and mu was not carried out^45^. As a result, there were cases where the number of fields was concealed to avoid taxation.
5. Mian-Ke. During the late Ming and early Qing dynasties, many wars of various sizes in Guizhou caused significant damage to agriculture, leaving the land barren and agricultural production greatly affected. In order to stabilize Guizhou, the Qing government took a series of positive measures to restore agricultural production. During the Kangxi, Yongzheng and Qianlong periods, reclamation was strongly encouraged, the Mian-Ke policy was implemented, and the proportion of Mian-Ke was relatively high with almost no upper limit^43^, resulting in the number of lands in the registers being too low.

According to the above analysis, it is believed that the main reasons for the severe underestimation of the data on cropland in Guizhou's register are the Yin-Ni and the Mian-Ke policy.

#### Cropland area correction

The data on cropland in the register is seriously underestimated due to the effects of the Yin-Ni and the policy Mian-Ke, so it should be corrected. Although Yin-Ni and Mian-Ke cropland data are difficult to quantify, both are closely related to the population. Regarding underestimating the amount of cropland in the Qing dynasty in Guizhou, the data on the number of fields in the register were corrected mainly based on the revised population and cropland per capita.

According to the per-capita cropland area before the liberation of Guizhou, the research results by Cheng Anyun and Song Fengjiao^49–51^, the per capita grain possession in the middle and late Qing dynasty was 250 kg, and the average grain yield was 1200 kg/hm^2^. It can be deduced from the cropland area required for the survival of Guizhou in the Qing period. Regarding historical records and the results of relevant studies, the cropland per capita in 1820 was 3.5 mu.

#### Cropland correction data validation

The cropland correction data is mainly verified by grain demand and labour supply.

1. Formula for calculating the calibration of grain requirements.
  1 $${D}_{F}=\frac{{D}_{T}*{P}_{A}}{n*(L*i-c)}$$

where ${D}_{F}$ is the area of cropland required based on food demand, ${D}_{T}$ is the revised population data, ${P}_{A}$ is per capita grain demand, *L* is unit grain yield, *i* is the multiple cropping index of crops, *n* is planting proportion of grain, *c* is the outflow of other grains.

The per capita food requirement is quoted from Wu Hui's research, based on 210 kg of food per person per year^52^. The proportion of grain planting and grain yield per unit was adopted from the research results of Cao Xue et al., with n ≈ 70%^53^. The unit grain yield and crop replanting index were based on the research results of Cheng Anyun et al.^50^, L = 1200 kg/hm^2^, i = 1.27.

(2) Formula for calculating the calibration of labour supply.
  2 $${D}_{L}=P*M*LC$$

where ${D}_{L}$ is the arable area of the labour force, *M* is the proportion of the labour force, and *LC* is the arable area per labour unit.

According to research results, the proportion of labourers in the Qing dynasty was about 32%^54^; the arable area per unit of labour was adopted from the research results of Cao Xue et al. with LC = 1.33 hm^2^. Shi Zhihong argues that the maximum amount of land ploughed by one man in the south during the Qing dynasty did not exceed 0.67 hm^2^^54^. Zhao Gang et al. argue that one cow was ploughed on thirty mu of land in the north, while the average southern one was a dozen mu^55^. In the middle and late Qing Dynasty, oxen ploughing techniques were introduced in Guizhou, and it is thought that the extent of cropland per unit of labour in Guizhou in 1820 should be set at 15 mu, or 1 hm^2^.

#### Cropland correction result

**Figure 2:**
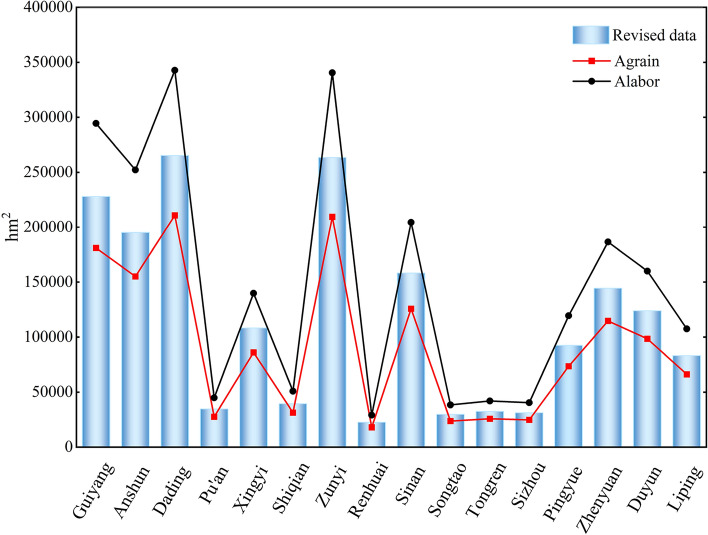
Results of Cropland data correction.

The results of the revised calibration of the cropland of Guizhou in 1820 are shown in Fig. 2.

As shown above, the cropland area in the sub-prefectures of Guizhou in 1820 was basically between the labour supply line and the subsistence line. It is converged with the subsistence line, consistent with the historical records.

**Figure 3:**
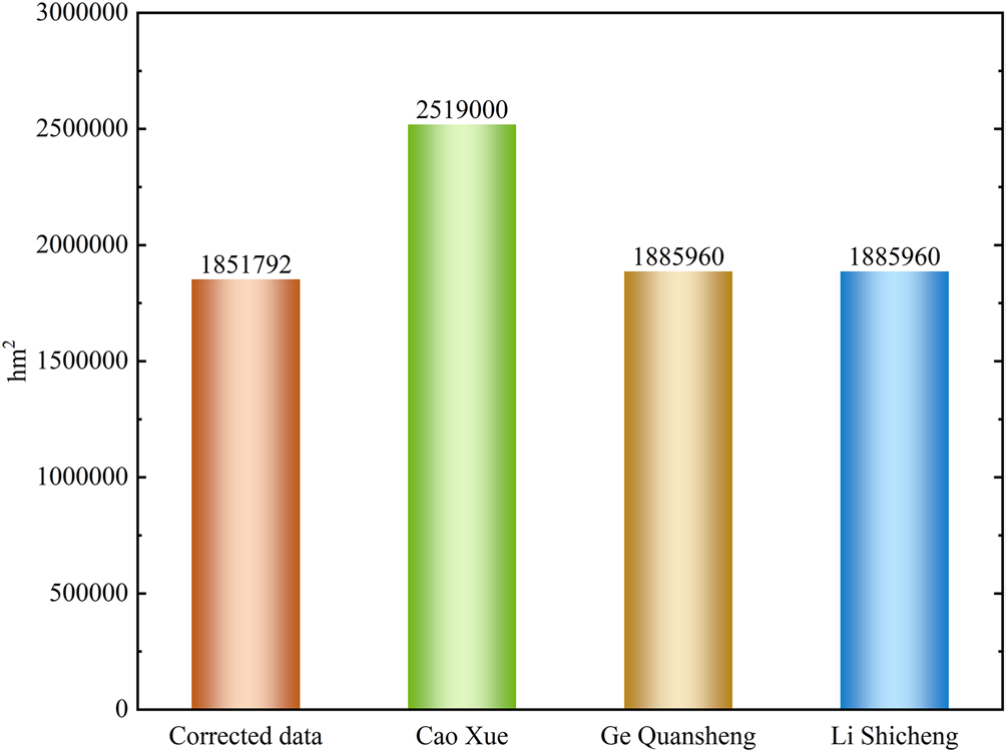
Comparison of cropland area correction.

The revised cropland area of Guizhou in 1820 is similar to the results of Cao Xue, Ge Quansheng and Li Shicheng (Fig. 3)^12,44,56^. It can be seen that the corrected data on the cultivated area are reliable and can be used in the study of the spatial pattern reconstruction for historical cropland. The corrected cropland area data for Guizhou are shown in Table 4.

**Table 4:** Correction data of cropland area.

Name	Area (hm^2^)	Proportion (%)	Name	Area (hm^2^)	Proportion (%)
Guiyang Fu	227,821	12.30	Sinan Fu	158,237	8.55
Anshun Fu	195,134	10.54	Songtao Ting	29,716	1.60
Dading Fu	265,214	14.32	Tongren Fu	32,440	1.75
Pu'an Ting	34,668	1.87	Sizhou Fu	31,202	1.68
Xingyi Fu	108,215	5.84	Pingyue Fu	92,367	4.99
Shiqian Fu	39,373	2.13	Zhenyuan Fu	144,370	7.80
Zunyi Fu	263,480	14.23	Duyun Fu	123,816	6.69
Renhuai Ting	22,535	1.22	Liping Fu	83,204	4.49

## Methods

The process of reclaiming cropland generally follows the principle of "first best, then worst", depending on the suitability of the land for farming^57^. Based on this principle, we select and quantify the dominant factors that affect the farmland's suitability for agriculture and establish a grid model for the spatial distribution of cropland^26^.

Both natural and human factors influence the cultivation of cropland. Due to the unique natural environment of Guizhou, slope, altitude, soil and water, and heat distribution will limit cropland distribution. The distribution of ethnic minorities and their farming habits will also affect the distribution of cropland, as reflected in the influence of the distribution of settlements on the distribution of cropland. In addition, anthropogenic factors such as population status, agricultural policies and the level of economic development will affect the macroscopic distribution of cropland to a certain extent. However, as their impact is difficult to quantify, the impact of anthropogenic factors on the spatial distribution pattern of cropland is not considered a focus of the study.

Considering the complexity of the natural environment and human factors in Guizhou, to make the simulation results closer to the historical reality, the relevant influencing factors on the suitability of land for agricultural production were divided into limiting and non-limiting factors according to the magnitude of their role in influencing the distribution of cropland. According to the characteristics of the natural environment, the level of agricultural production and the socio-economic development of the study area, slope, altitude and soil type are selected as limiting factors limiting the distribution of cropland. At the same time, climate production potential, soil organic matter content, distance from rivers and distance from settlements was used as non-limiting factors to construct calculation models for the degree of land reclamation and reclamation rate and reconstruct the spatial distribution pattern cropland in 1820 of Guizhou.

### Parameter quantization

**Figure 4:**
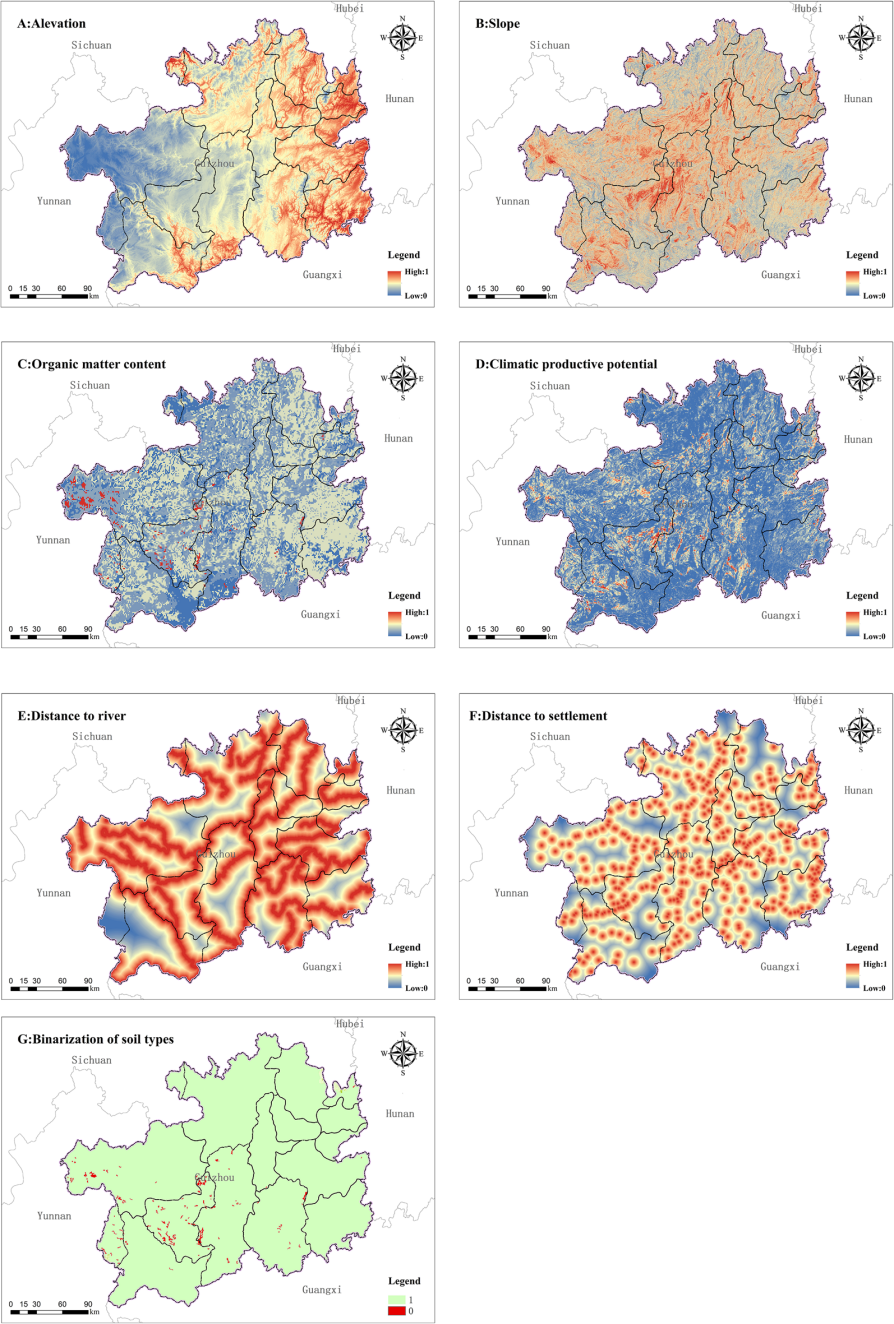
Spatial quantification of the arableability factor (map created using ArcGIS 10.8).

The study selected elevation, slope, river, soil type, soil organic matter content, and settlement distribution as factors influencing the spatial pattern distribution of historical cropland. In order to eliminate dimensional interference, each factor needs to be normalized to a final value in the range [0,1], with a value of 1 being the most suitable for land cultivation and a value of 0 being the least suitable. The spatial quantification of the arableability factors is shown in Fig. 4.

1. Elevation standardization

According to the analysis of the altitude distribution of cropland in Guizhou in 1980, the cropland at an altitude of 2200 m was taken as the upper limit of cropland in Guizhou during the Qing Dynasty.

3 $${NE}_{ij}=\frac{max\left({e}_{ij}\right)-{e}_{ij}}{max\left({e}_{ij}\right)}$$

where ${NE}_{ij}$ is the elevation normalized value, ${e}_{ij}$ is the average elevation of the grid *i* in the *j*th partition, ${max}_{(ij)}$ is the maximum elevation in the *j*th partition.

(2) Slope standardization

Based on the research and analysis of the slope of cropland in Guizhou in 1980^58,59^, and taking into account the cultivation situation in Guizhou during the historical period, it is considered that cropland with a slope of 30° was the upper limit of cultivation in Guizhou during the Qing Dynasty.

4 $${\mathrm{NS}}_{ij}=\frac{max\left({s}_{ij}\right)-{s}_{ij}}{max\left({s}_{ij}\right)}$$

where ${NS}_{ij}$ is the slope normalization value, ${max}_{(ij)}$ is the maximum slope value in the *j* region.

(3) Standardization of organic matter content
  5 $$N{\mathrm{Q}}_{ij}=\frac{max\left({O}_{ij}\right)-{O}_{ij}}{max\left({O}_{ij}\right)}$$

where ${NQ}_{ij}$ is the normalized value of organic matter content, ${max(o}_{ij})$ is the maximum value of the corresponding indicator.

(4) Standardization of climatic productivity potential
  6 $${NC}_{ij}=\frac{{C}_{ij}}{max({C}_{ij})}$$

where ${NC}_{ij}$ is the normalized value of climatic production potential, ${max}_{(Cij)}$ is the maximum value of the corresponding indicator.

(5) Standardization of distance to rivers

The 1820 distribution of rivers in Guizhou provided by CHGIS was used as the base study data, and 1500 m was taken as the buffer radius, normalized using the maximum negative normalization method.

(6) Standardization of distance from settlements

In agricultural societies, cropland gradually expands outwards with the settlement as the center. The cultivation radius for walking is 500 m, and 1000 m is more appropriate. The 1820 Guizhou village settlement provided by CHGIS was used as the base study data for the settlement. The center of the settlement was used to make a buffer radius of 500 m from the settlement, which was normalized using the maximum negative normalization method.

(7) Binarization of soil types

In this study, soil types were binarized, the soil types suitable for cultivation were assigned a value of 1, and the soil types unsuitable for cultivation were assigned a value of 0.

### Reconstruction the spatial pattern of cropland

After normalizing the limiting and non-limiting factors affecting the spatial distribution of cropland, a model of the spatial distribution of cropland with a spatial resolution of 90 m × 90 m was constructed.

7 $${W}_{ij}=\prod_{x=1}^{n}{R}_{ij}\sum_{y=1}^{h}{D}_{x}{A}_{x}{\omega }_{i}$$

where ${W}_{ij}$ is the degree of land reclamation of grid *i* in zone *j*, ${R}_{ij}$ is the reclamation restriction factor in zone *j*, ${D}_{x}$ is the unrestricted reclamation factor, ${A}_{x}$ is the weight of the unrestricted factor. The geometric mean model used in this model removes land unsuitable for cultivation. The reclamation of the grid reaches 0 when the restriction factor in the grid exceeds the upper limit.

The formulae for calculating the cropland area ${C}_{ij}$ and the reclamation rate ${FR}_{ij}$ for grid *i* in the *j* area. As is shown follows:

8 $${C}_{ij}={T}_{ij}\times \frac{{W}_{ij}}{{\sum_{i=1}^{n}W}_{ij}}$$

9 $${FR}_{ij}=\frac{{C}_{ij}}{{S}_{ij}}$$

where ${C}_{ij}$ is the cropland area of grid *i*, ${T}_{ij}$ is the total area of cropland in zone *j*, ${FR}_{ij}$ is the reclamation rate of grid *i* in zone *j*, ${S}_{ij}$ is the area of each grid, which is 8100 m^2^.

Because of the set reconstruction grid size of 90 m × 90 m, some grid reclamation rates may be greater than 1 when performing calculations. Although the settlement rate in the historical period generally does not exceed 90%, because the 90 m × 90 m high resolution is used for the study, the grid reclamation rate maybe 100%, so the maximum value of the settlement rate is controlled to 100%. When the settlement rate of a grid is greater than 1, it is assigned a value of 1, and the excess cropland is reallocated according to the above formula until all grids are ${\mathrm{FR}}_{\mathrm{ij}}$ ≤ 1.

## Results and discussion

### Results analysis

#### Results of spatial pattern reconstruction of cropland

**Figure 5:**
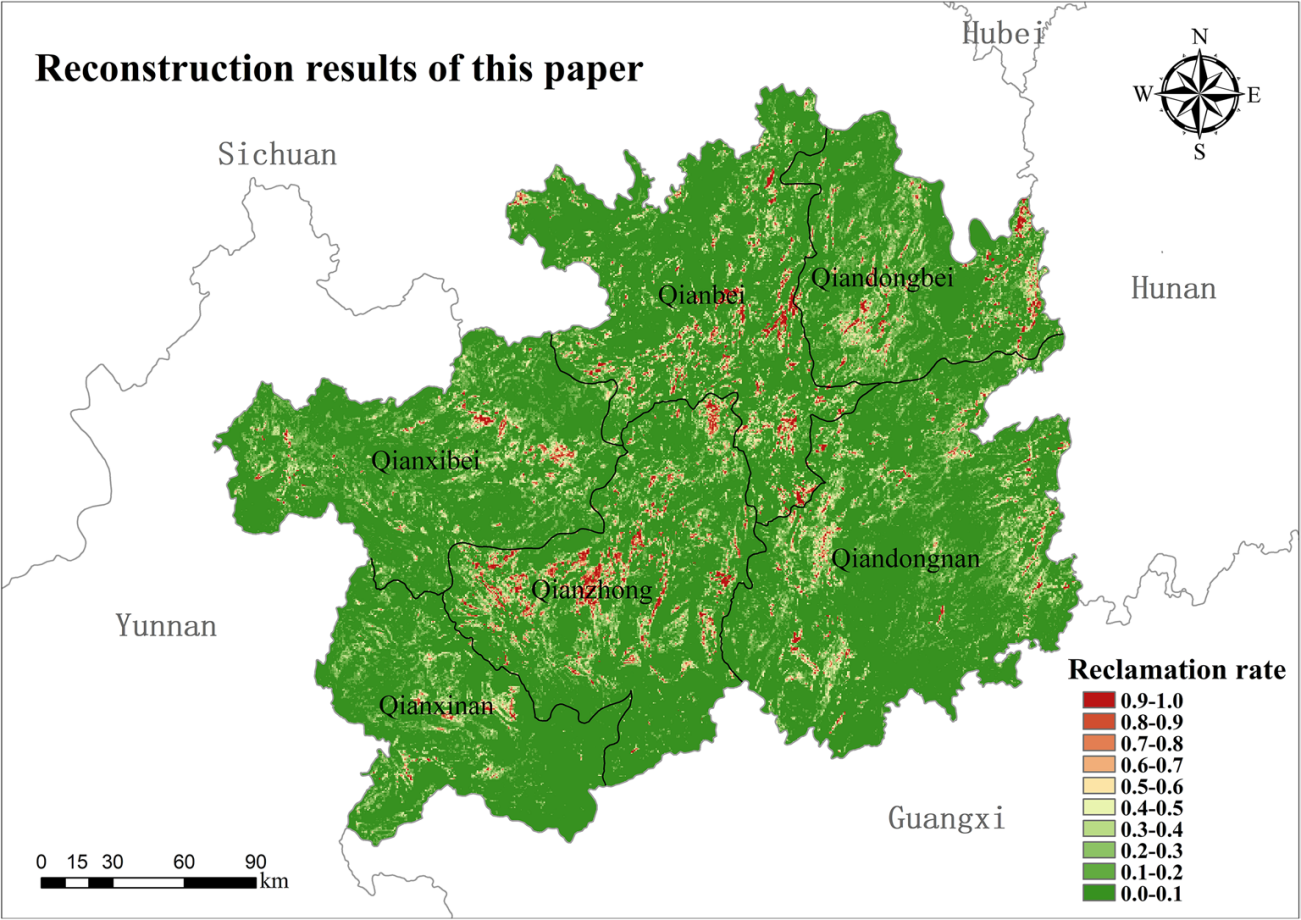
Reconstruction results of the cropland pattern in this paper (map created using ArcGIS 10.8).

The results of the reconstruction of cropland distribution in Guizhou in 1820 at 90 m × 90 m resolution are shown in Fig. 5. The results of the reconstruction of the cropland distribution at 90 m × 90 m resolution in Guizhou in 1820, the results of the spatial distribution of cropland were graded into statistics using 10% as the interval of change in the settlement rate, as shown in Table 5.

**Table 5:** Reconstruction result settlement rate interval statistics.

Name	Interval distribution of reclamation rate (%)									
	0–10	10–20	20–30	30–40	40–50	50–60	60–70	70–80	80–90	90–100
Qianbei	71.4	11.18	3.68	4.09	2.82	1.95	1.33	0.91	0.7	1.94
Qiandongbei	63.41	16.43	8.12	4.44	2.65	0.82	1.26	0.88	0.58	1.41
Qiandongnan	72.31	13.62	6.07	3.27	1.89	1.11	0.23	0.56	0.5	0.44
Qianzhong	66.44	11.3	6.89	4.54	2.33	1.42	2.04	1.4	1.57	2.07
Qianxinan	78.02	11.64	5.06	2.4	1.23	0.67	0.24	0.33	0.21	0.2
Qianxibei	70.38	15	7.22	3.02	1.71	0.98	0.56	0.14	0.61	0.38
Guizhou	70.41	13.1	6.09	3.64	2.12	1.21	0.93	0.71	0.72	1.08

#### Analysis of the distribution of cropland

Due to the limitations of the natural environment and the historical level of agricultural production, in 1820, the proportion of land used for cultivation in Guizhou was relatively small, with only 30% of the grid having cropland distribution. Cropland was mainly concentrated in the central region of Qianzhong District (Anshun and Guiyang Fu), the southern part of Qianbei District (Pingyue Fu and the southern part of Zunyi Fu), the western part of the Qiandongbei District, the central and eastern parts of the Qiandongbei District. Most of the altitudes in Qianzhong are from 800 to 1000 m. The main landforms are mountainous hilly depressions and mountainous hilly basins with relatively flat terrain. The region has a warm and humid climate, abundant water resources, fertile soil and concentrated cropland, which is very conducive to the growth of crops. It is the birthplace of Guizhou's agrarian civilization and the main gathering area, the main reclamation area for agricultural cultivation and the main food and cash crop production area in Guizhou's historical period. With its relatively good irrigation conditions, fertile land and humid climate, Qianbei was an important rice-producing area and a major source of taxation during the Qing Dynasty. Qiandongnan District is rich in water resources, with rivers crisscrossing the region and three major rivers, the Qingshui River, the Maoyang River and the Duliu River. The area has good irrigation conditions, fertile soil and a warm and humid climate throughout the year. The heat is abundant, with an active cumulative temperature of ≥ 10° of about 4500–5400 °C and annual precipitation of about 1200–1500 mm. Its warm and humid climate is extremely beneficial to the growth of crops. The central and eastern parts of the Qiandongbei District have better water and heat conditions, especially in the low-altitude river valleys, where heat is abundant and the growing season is long. Tongren and Sinan Fu, due to their early historical development and the vigorous promotion of the culture of the Central Plains, agricultural farming techniques and water conservancy projects have been well developed in the region, resulting in a high rate of cropland settlement in the region.

#### Analysis of cropland reclamation intensity and difference

**Figure 6:**
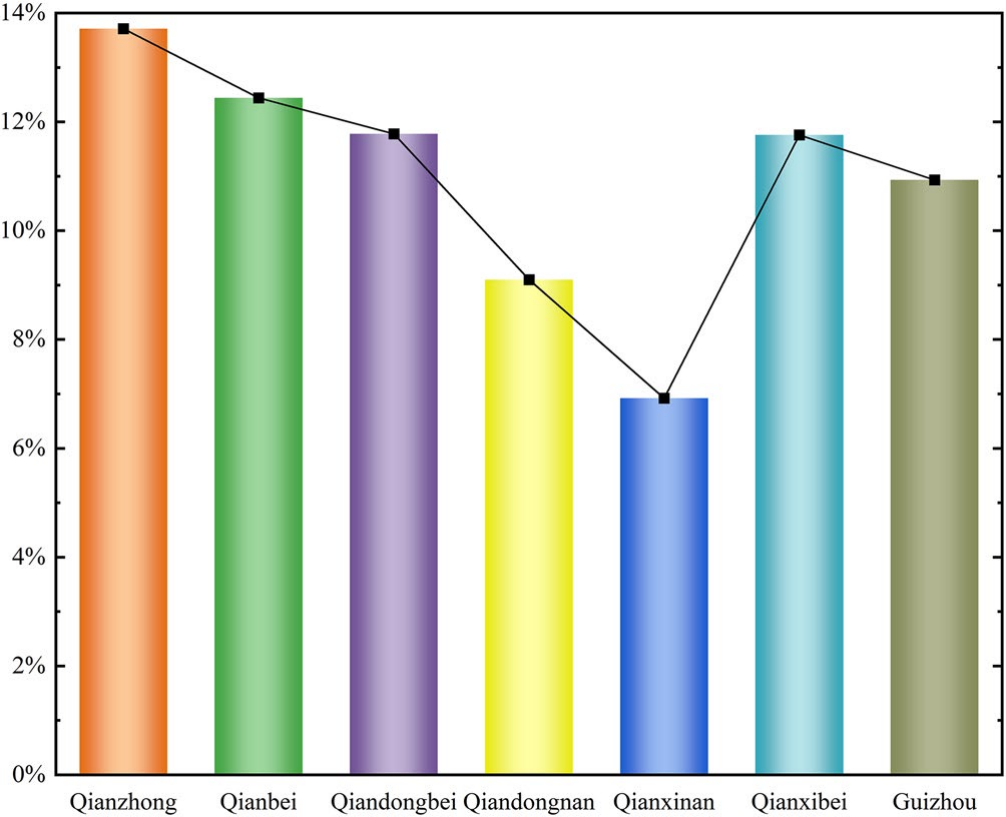
Average reclamation rate by district.

The average rate of reclamation is shown in Fig. 6. The overall average settlement rate in Guizhou in 1820 was 10.93%, with only 4.65% of the grids with a settlement rate ≥ 50%, 24.95% of the distribution of grids with a 10% ≤ reclamation rate < 50%, and up to 70.41% of the grids with a settlement rate of less than 10%. The average settlement rate in Qianzhong District was the highest at 13.71%, followed by 12.44% in Qianbei District, 11.78% in Qianbei District, and 11.76% in Qianxi District, 9.1% in Qiandongnan District and the smallest in Qianxinan District with an average settlement rate of 6.92%.

**Figure 7:**
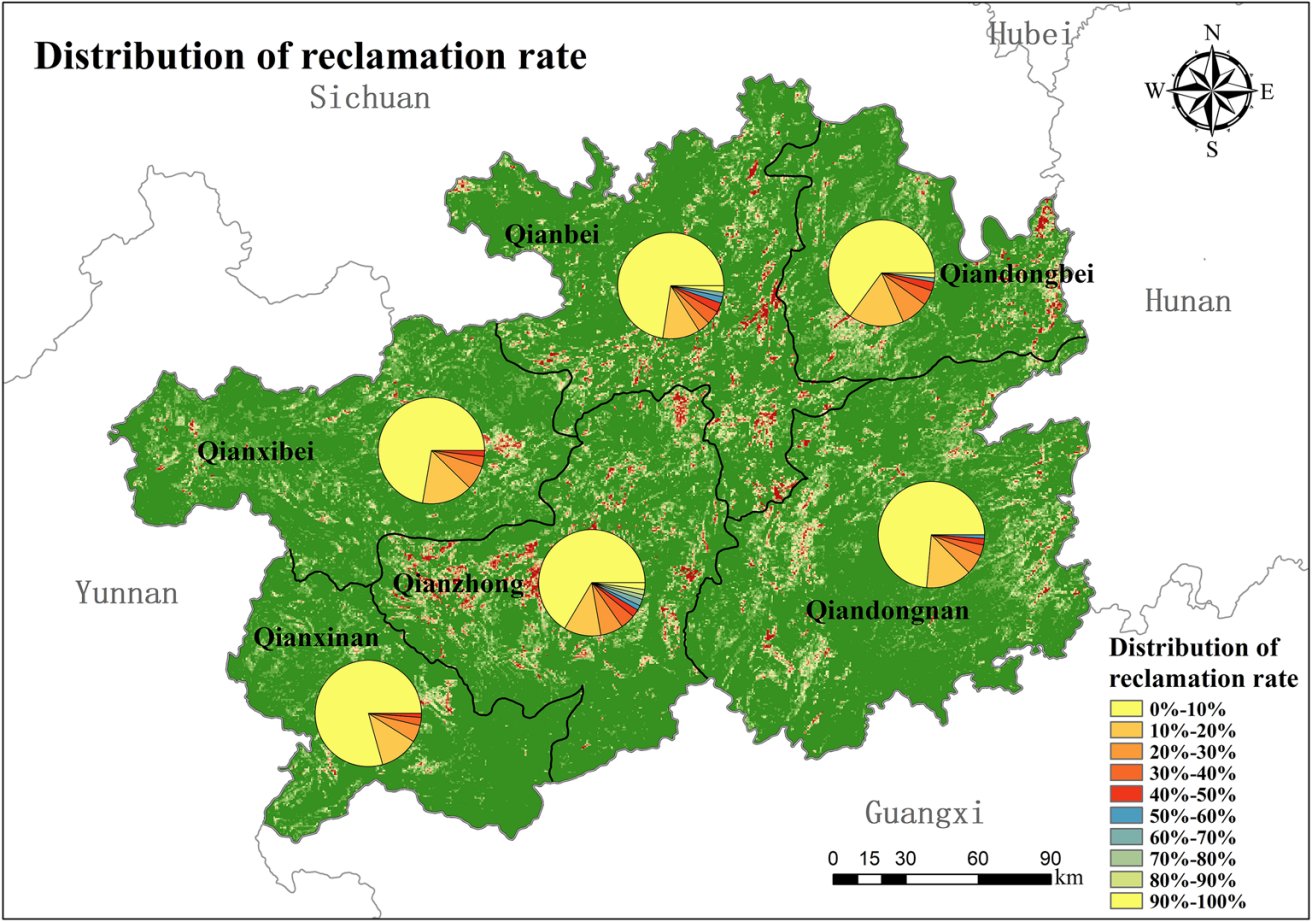
Percentage of settlement rate distribution intervals by district (map created using ArcGIS 10.8).

Due to the constraints of topography, elevation and other related factors, there is significant variability in the regional distribution of land reclamation intensity in the study area (Fig. 7). Among them, Qianzhong District has the highest land reclamation intensity, with 8.5% of cropland with a reclamation rate ≥ 50%, 25.06% of grid distribution with 10% ≤ reclamation rate < 50%. This is inextricably linked to the high population density of the Qianzhong region, which was Guizhou's political and cultural center during the Qing Dynasty, and the topography, climate, and soil suitable for agricultural production. It is followed by Qianbei District, with 6.83% of the grids with a reclamation rate ≥ 50%. The least intensity of reclamation was found in Qianxinan District, with only 1.65% of the grid with a reclamation rate ≥ 50%.

### Discussion

#### Comparative validation analysis of reconstruction models and reconstruction results

**Figure 8:**
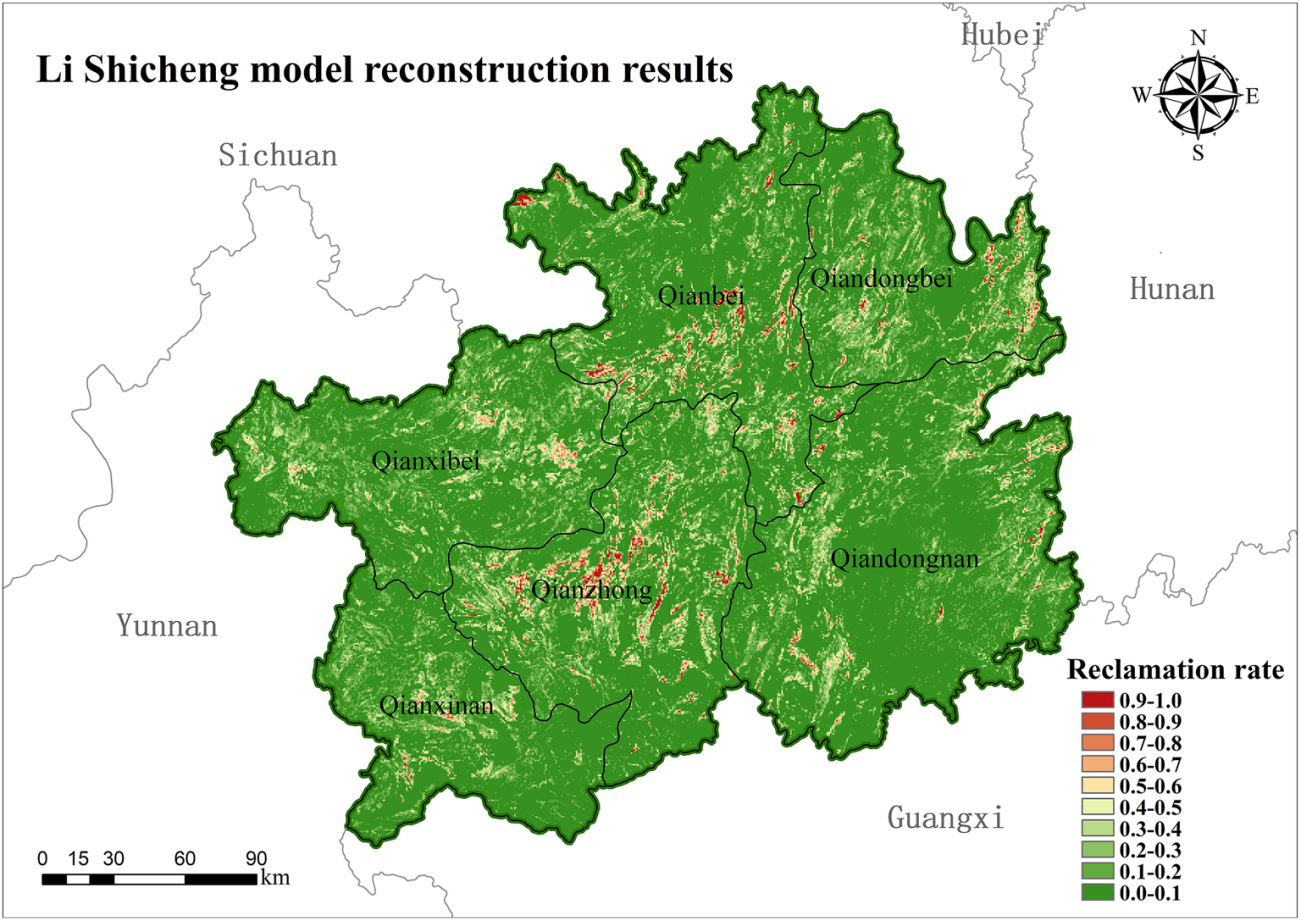
Reconstruction results of the cropland spatial pattern in 1820 by Li Shicheng model (map created using ArcGIS 10.8).

In order to verify the rationality of the model constructed by the study, the reconstructed results were compared and analyzed with the results of the spatial pattern distribution of 90 m × 90 m cropland in Guizhou in 1820 (Fig. 8) as reconstructed by Li Shicheng for his model of the spatial distribution pattern of cropland in southwest China during the Qing Dynasty.

From the overall spatial distribution of the reconstruction results, the grid-based reconstruction model constructed in this paper is generally consistent with the spatial distribution pattern of cropland in Guizhou in 1820 as simulated by the reconstruction model of Li Shicheng, which is mainly distributed in the northwestern part of Anshun Fu, Guiyang Fu, Pingyue Fu, Duyun Fu and the southern part of Zunyi Fu. Although there is a good agreement in distribution, the reconstructed results from the two models have some differences in the extent and intensity of settlement. The results of the reconstructed model by Li Shicheng show that about 66% of the grids have no cropland and 34% of the grids have cropland distribution. The results of the reconstruction model in this paper show that only about 29% of the grids in Guizhou had cropland distribution in 1820, and about 71% of the grids did not have cropland distribution. The distribution of cropland reconstructed by the model of Li Shicheng was more extensive, and the reconstruction results of each subdistrict showed the same trend.

**Figure 9:**
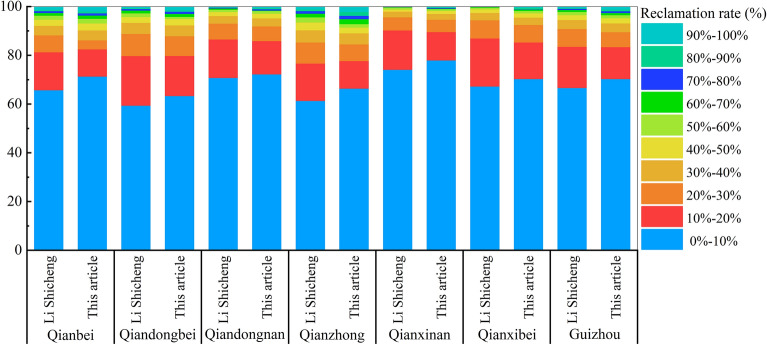
Distribution of reclamation rate intervals by district.

In terms of the intensity of settlement of the reconstructed cropland, the intensity of the settlement rate of the model reconstructed in this paper is higher than that of the reconstructed result of the Li Shicheng model, and the distribution of cropland is more concentrated (Fig. 9). The model reconstruction results in this paper show that 4.65% of the grids with a reclamation rate ≥ 50%, 24.95% of the distribution of grids with 10% ≤ reclamation rate < 50%, and up to 70.41% of the grids with a reclamation rate of less than 10%. In the results of the cropland distribution in Guizhou in 1820 reconstructed by Li Shicheng's model, only 3.39% of the grids with a settlement rate ≥ 50%, 29.8% of the distribution of grids with 10% ≤ reclamation rate < 50%, and 66.73% of the grids with a settlement rate of less than 10%.

**Figure 10:**
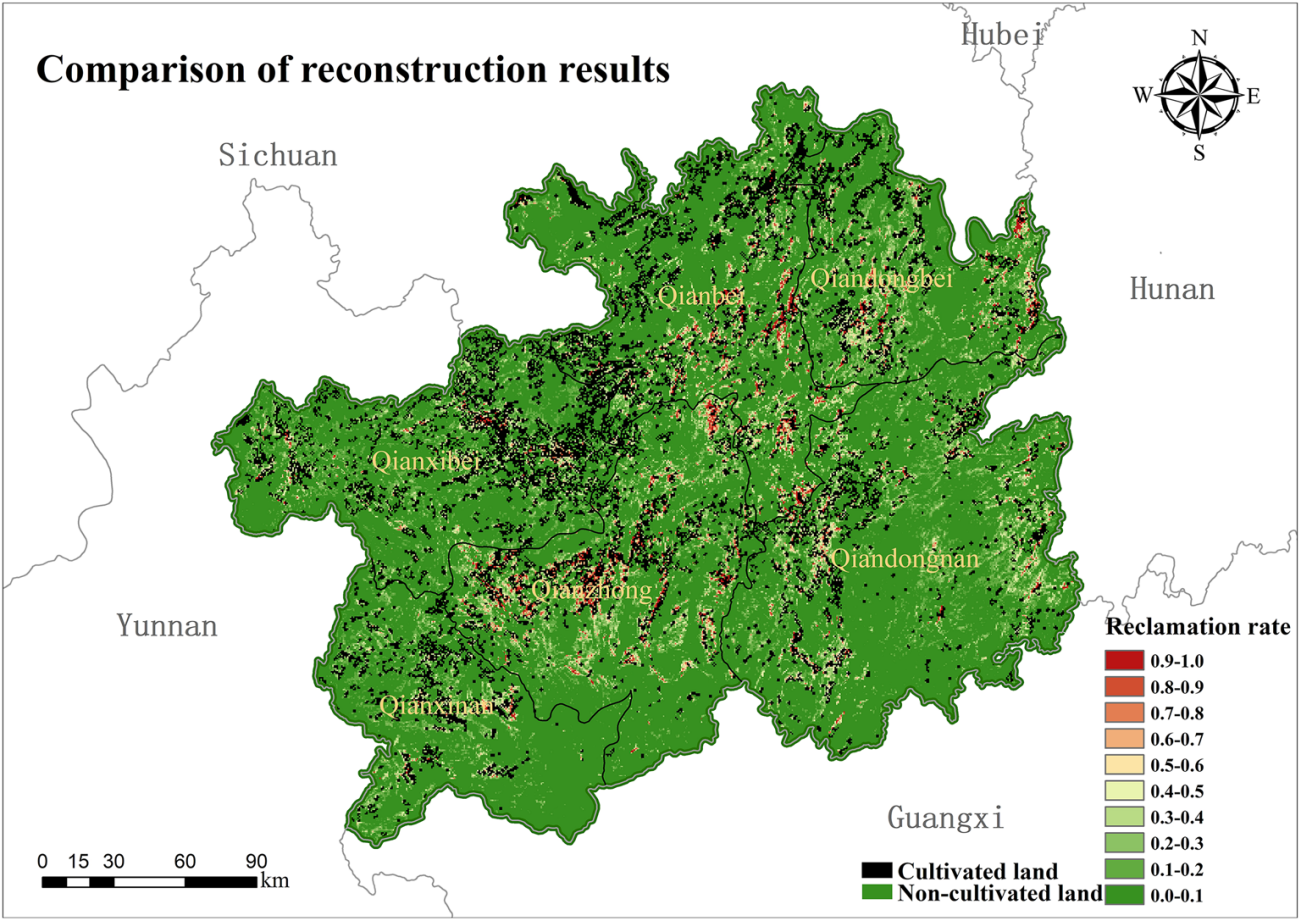
Comparison of cropland reconstruction results (map created using ArcGIS 10.8).

The distribution of cropland in Guizhou in 1820, reconstructed by Yang Xuhong's model, was overlaid with the distribution of cropland reconstructed by the model in this paper, as shown in Fig. 10. The black is the result of the cropland distribution reconstructed by Yang Xuhong's model. The bottom map's red and pale yellow are the cropland distribution area reconstructed by the model in this paper. Although there are slight differences in their spatial distribution, the general trend is the same, with cropland concentrated in the Anshun Fu, Guiyang Fu, Pingyue Fu, northwestern Duyun Fu and the southern area of Zunyi Fu. As the model in this paper is based on the spatial network distribution of potential cropland in the historical period, Yang Xuhong's reconstruction model is based on the "bottom-up" method of reconstructing the spatial pattern of cropland in the historical period, so there are some differences between the two reconstruction results. However, the overall distribution pattern of cropland is still consistent.

#### Evaluation of reconstruction models and reconstruction results

As there is a considerable lack of information and maps on historical cropland data, especially those with spatial attributes, it is difficult to directly verify and evaluate the rationality and accuracy of the reconstruction results of the spatial pattern of cropland in the historical period. To verify the applicability of the reconstruction model in the reconstruction of the spatial pattern of cropland in karst areas and to evaluate the accuracy of the reconstruction results. A comparative validation method was used to test and evaluate the reconstruction model and the reconstruction results of the spatial distribution pattern of cropland.

The analysis was verified by comparing the reconstructed model and the reconstruction results with those of Li Shicheng and Yang Xuhong. It can be seen that although there are some differences in the reconstructed results of the spatial distribution pattern of cropland, the overall spatial distribution is consistent and can reflect the general pattern of cropland distribution in Guizhou in 1820. The model in this paper takes more into account the constraints of the natural environment in the karst region on the spatial distribution of cropland and reconstructs the spatial distribution of historical cropland at a high resolution of 90 m × 90 m. Therefore, the reconstructed model and the results of this paper can more objectively reflect the distribution of cropland in karst areas during the historical period and are more applicable to Guizhou during the Qing Dynasty when the productivity level was low.

#### Follow-up studies

Based on the historical documentary data, the cropland data in the book were revised, and the complexity of the natural environment and human factors, the suitability of cultivation and the rules of concentrated and continuous cultivation in karst areas were taken into account. A zone-synchronous reconstruction model of the spatial pattern of historical cropland was established, and the spatial distribution of cropland in Guizhou in 1820 was reconstructed under a 90 m × 90 m spatial grid. First, in terms of accuracy in reconstructing the spatial pattern of arable land, there is still room for further improvement. Studies with higher accuracy can be applied to the karst mountains. Second, because anthropogenic factors that influence cropland distribution are difficult to quantify, only the anthropogenic factor of settlement distribution was chosen to reconstruct the spatial pattern of cropland in the historical period. The influence of anthropogenic factors on cropland distribution in the historical period was less considered. In the subsequent study, the influence of anthropogenic factors on the distribution of cropland should be strengthened to reconstruct a more accurate pattern of cropland distribution in the historical period. In addition, the natural environment in karst areas is fragile, the conflict between man and land has been more acute, the response to climate change is sensitive, and the ability to resist human activities and natural disturbances is significantly lower than in other areas. Strengthening the reconstruction of historical land use in karst areas can reproduce the historical land-use situation and provide a database for studying ecological environment change and regional climate change, especially for the inversion study of karst rock desertification in karst areas. Therefore, comprehensive applied research on land use reconstruction work in karst areas should be increased at a later stage.

## Conclusion

During the Qing Dynasty, Guizhou's recorded population and cropland data were too low, mainly due to the Yin-ni of minority fields and the Qing government's vigorous implementation of the "Tu-Di-Mian-Ke" policy in Guizhou. In 1820, the total area of revised cropland in Guizhou was 1,851,792 hm^2^, with the highest proportion of 14.32% in Dading Fu and the lowest in Songtao Ting at 1.6%. Only 30% of the grid in Guizhou has cropland distribution. It is mainly concentrated in the central part of Qianzhong District (Anshun and Guiyang Fu), the southern part of Qianbei District (Pingyue Fu and south of Zunyi Fu), the western part of Qiandongnan District, the central and eastern parts of the Qiandongbei District. The overall average reclamation rate in Guizhou was 10.93%, among which the highest land reclamation intensity was found in Qianzhong District, with 8.5% of the grids with a reclamation rate ≥ 50%, and the smallest in Qianxinan District, with only 1.65% of the grids with a reclamation rate ≥ 50%. The results of this study are consistent with the reconstructed results of the model of Li Shicheng and Yang Xuhong in terms of overall spatial distribution, and both can reflect the general pattern of cropland distribution in Guizhou in 1820, but there are some differences in the results of settlement rate. The reconstruction results are reasonable and can objectively reflect cropland distribution in karst areas during the historical period. The reconstruction model is suitable for karst areas with low productivity levels.

## Data Availability

The datasets generated and/or analyzed during the current study are not publicly available due project requirements but are available from the corresponding author on reasonable request.
